# Description of a *Zostera marina* catalase gene involved in responses to temperature stress

**DOI:** 10.7717/peerj.4532

**Published:** 2018-03-26

**Authors:** Yu Zang, Jiao Liu, Xue Xi Tang, Bin Zhou

**Affiliations:** 1 College of Marine Life Science, Ocean University of China, Qingdao, China; 2 Laboratory for Marine Ecology and Environmental Science, Qingdao National Laboratory for Marine Science and Technology, Qingdao, China; 3 Institute of Oceanology, Chinese Academy of Sciences, Qingdao, China

**Keywords:** CAT, Temperature stress, mRNA expression, Enzyme activity

## Abstract

Catalase (CAT) is an antioxidant enzyme that plays a significant role in cellular protection against oxidative damage by degradation of hydrogen peroxide to oxygen and water. In the present study, the complete CAT cDNA sequence of *Zostera marina* was identified through expressed sequence tags (EST) analysis and the rapid amplification of cDNA ends (RACE) technique. The nucleotide sequence of *Zm*CAT cDNA consisted of 1,816 bp with a 1,434 bp open reading frame (ORF), encoding a polypeptide of 477 amino acid residues, which possessed significant homology to other known plant CATs. The molecular mass of the predicted protein was 55.3 kDa with an estimated isoelectric point of 6.40. Phylogenetic analysis showed that *Zm*CAT was closely related to CAT from gramineous species. In response to temperature stress, H_2_O_2_ and MDA contents in *Z. marina* increased significantly with cold stress (<10 °C) and heat stress (>25 °C). *Zm*CAT expression was significantly upregulated at temperatures from 5 to 10 °C and then gradually downregulated, reaching its lowest expression at 30 °C. Recombinant *Zm*CAT protein exhibited strong antioxidant activity over a wide temperature range, with the highest r*Zm*CAT activity observed at 25 °C and a higher relative activity retained even with heat stress. All these results indicated that *Zm*CAT was a member of the plant CAT family and involved in minimizing oxidative damage effects in *Z. marina* under temperature stress.

## Introduction

Seagrass meadows, covering ∼0.1–0.2% of the global ocean floor, are highly productive ecosystems that provide key ecological services to the marine environment (Erftemeijer & Robin Lewis III, 2006; Orth et al., 2006). *Zostera marina* (or eelgrass), one of the most widespread seagrass species throughout the temperate northern hemisphere, plays an important ecological engineering role by influencing sediment stabilization, carbon cycling, and food web structure (Fourqurean et al., 2012; Hemminga & Duarte, 2000). In recent decades, *Z. marina*, as well as other species of seagrass is declining as a result of  aquatic environmental changes, which will become more noticeable with sustained global climate change (Orth et al., 2010; Short et al., 2011). Temperature has long been recognized as a major environmental factor affecting the biogeographical distribution and health of seagrass meadows (Bulthuis, 1987; Ralph, 1998). Furthermore, recent large-scale seagrass populations have been precipitously decimated worldwide from historical abundances, caused by extreme summer heat waves (Moore, Shields & Parrish, 2014; Thomson et al., 2015), suggesting the question regarding how global climate change might aggravate this decline.

Temperature stress is known to accelerate the generation of reactive oxygen species (ROS), such as singlet oxygen (^1^O_2_), hydrogen peroxide (H_2_O_2_), superoxide radical (O_2_⋅), and hydroxyl radicals (⋅OH), thereby leading to oxidative damage (Potters et al., 2007; Jena et al., 2013; Mittler, 2002). ROS production is a necessary process that can occur naturally and is related to both respiratory and photosynthetic metabolism, representing normal aerobic metabolism of plants. However, under abiotic stressful situations, homeostasis between the production and scavenging of ROS is altered and causes ROS accumulation (Wang et al., 2010). Excessive accumulation of ROS can cause oxidative damage through increasing lipid peroxidation (LPO), protein oxidation and degradation, and double-strand DNA breakage (Matés, 2000). To maintain homeostasis and protect against oxidative stress, plant cells have evolved antioxidant defense mechanisms, such as antioxidant enzymes, including catalase (CAT), superoxide dismutase, ascorbate peroxidase, glutathione peroxidase, glutathione reductase, and glutathione peroxidase (Hasanuzzaman, Nahar & Fujita, 2013). Among these enzymes, CAT plays a critical role in antioxidant defense pathways by efficiently catalyzing, in peroxisomes and glyoxysomes, the conversion of two molecules of H_2_O_2_ to two molecules of H_2_O and an O_2_(2H_2_O_2_ → 2H_2_O +O_2_) through the transfer of two electrons, thus counteracting H_2_O_2_ toxicity (Kashiwagi et al., 1997; Srivastava et al., 2012). Regulation mechanisms of CAT have been investigated at the transcriptional and enzymatic levels, which demonstrated that mechanisms of plant adaptions to heating and chilling stress might be positively correlated with CAT responses in maize (Scandalios, Acevedo & Ruzsa, 2000) cucumber (Gao, Qin & Yu, 2009) olive (Cansev, Gulen & Eris, 2011), broccoli (Lin, Huang & Lin, 2010) and banana (Figueroa-Yáñez et al., 2012).

The recent development of molecular resources for different seagrass species has provided important new insights into the knowledge of the transcriptional control of temperature stress responses (Franssen et al., 2014; Gu et al., 2012; Marin-Guirao et al., 2017). Our first attempts have already been carried out in the MnSOD gene of *Z. marina* and found that it was beneficial in minimizing oxidative damage effects of temperature stress (Liu et al., 2016). For seagrass, CAT activity has been detected in many species, such as *Zostera japonica* (Lin et al., 2016) and *Posidonia oceanica* (Sureda et al., 2008), but there have been no reports regarding detailed analysis and characterization of genes encoding CAT from any species of seagrass except for a partial CDS deposited in GenBank from *Cymodocea nodosa*. Study of the structural characteristics and antioxidant responses of *Zm*CAT will help us to determine the functional roles of *Zm*CAT in response to temperature stress. Thus, the purposes of this study were to: (1) clone the full-length cDNA sequence of *Zm*CAT and analyze the evolution of *Zm*CAT among other species (2) investigate the effects of temperature stress on H_2_O_2_ and malondialdehyde (MDA) contents in *Z. marina* (3) validate mRNA expression and recombinant protein activity of *Zm*CAT in response to temperature treatments.

## Materials and Methods

### Plant material

Eelgrass vegetative shoots with attached roots were collected from the subtidal zone in Huiquan Bay (36°05′19.4″N, 120°34′46.2″E; Qingdao City, China) The research permit was issued to the College of Marine Life Science, Ocean University of China (Permit number: 2013OUCOLS0618). Plants were maintained in plastic boxes and transported within 2 h to the laboratory. Plant surface epiphytes and the older leaves were removed and individual shoots planted in plastic hydroponic planting baskets (100 mm inner diameter), which were filled with washed and sterilized silt (sediment particle size <63 µm) from a collection site in Huiquan Bay. The baskets were placed at aquaria bottoms and provided aerated filtered natural seawater at a constant temperature of 15 °C and allowed to acclimatize for 7 d. Fluorescent lamps provided an overhead light source with an applied light intensity of 150 µmol m^−2^ s^−1^ and light/dark regime of 12/12 h. Healthy shoots of *Z.marina* were used for the following experiments.

### Temperature treatments

A total of 30 eelgrass plants were employed for the temperature stress stimulation treatment, with plants were randomly divided into 6 groups. Five eelgrass plants were evenly spaced and kept upright in each of 6 glass aquariums (195 L). Temperature treatments were at 5, 10, 15, 20, 25, and 30 °C for 96 h, maintained by an automatic water temperature regulator (RESUN, 800W/S2TS2500) in each aquarium. During the experiment, aquaria seawater was adequately aerated and circulated using multifunctional submersible pumps to ensure mixing and homogeneous temperature within each aquarium. All experimental seawater was completely changed daily to avoid nutrient limitations, and the salinity and water temperature was checked daily. After treatment, leaf samples (*n* = 5) were cut into 100 mg (fresh weight, FW) sections after 5 cm segment from the leaf base, flash frozen in liquid nitrogen and rapidly stored at −80 °C until further use.

### Total RNA extraction and cDNA synthesis of *Zm*CAT

Total RNA was extracted from fresh leaf samples (50 mg) of eelgrass with RNA Extract Kit (Aidlab Biotechnologies Ltd., Beijing, China) according to the manufacturer’s instructions. The quality and integrity of total RNA were assessed by a NanoDrop (Thermo Fisher Scientific, Delaware, USA) and 1% agarose gel electrophoresis. The first-strand synthesis was carried out using the RQ1 RNase-free DNase I (Promega Corp., Madison, WI, USA) treated raw RNA (4 µg) as template and adaptor primer-oligo (dT) as primer (Table S1). The reaction were performed at 55 °C for 1 h and terminated by heating at 70 °C for 5 min. Products from this reaction were subsequently stored at −80 °C.

### Expressed sequence tags analysis and cloning of full-length *Zm*CAT cDNA

BLAST analysis of all ESTs sequences from the *Z. marina* cDNA library in National Center for Biotechnology Information (NCBI) showed that an EST of 487 bps sequence (Accession No. AM768877) showed high similarity to identified CATs from *Musa acuminata subsp. Malaccensis* (accession number XM009402721); thus, this EST sequence was selected for further cloning the full-length cDNA sequence of *Zm*CAT. Two gene specific primers, *Zm*CAT-Race-F_1_ and *Zm*CAT- Race-F_2_ (Table S1), were designed to amplify the sequence of *ZmCAT* cDNA by the rapid amplification of cDNA ends (RACE) technique. PCR products were gel-purified and cloned into the T-A cloning vector pMD19-T simple (Takara Bio Inc. Shiga, Japan). After transformation into competent cells of *E. coli* DH5 *α*0, positive recombinants were recognized by blue-white selection on ampicillin-containing Luria-Bertani plates and white colonies subsequently screened by PCR with M13-47 and RV-M primers (Table S1). Positive clones were sequenced on an ABI Prism™ 3730 automated DNA sequencer (Thermo Fisher Scientific Inc., Pittsburgh, PA, USA).

### Bioinformatical analysis of nucleotide and amino acid sequences

The nucleotide and deduced amino acid sequences of *Zm*CAT cDNA were analyzed using the BLAST software of NCBI and signal peptides predicted with the SignalP 4.1 Server. Besides, functional domains and signature motifs were identified using the normal mode of the Simple Modular Architecture Research Tool (Letunic, Doerks & Bork, 2012) and PROSITE database. Cellular localization of the inferred amino acid sequence was carried out with the PSORT Prediction tool of the PSORT WWW Server. A multiple sequence alignment was generated using the Clustal Version 2.0 program (Larkin et al., 2007). A phylogenetic tree was then produced by the neighbor-joining (NJ) approach of the MEGA 6.0 program (Tamura et al., 2013), using 5,000 bootstrap replications.

### Analysis of the H_2_O_2_ and MDA contents

Leaf samples were ground under liquid nitrogen and homogenized in 0.9  mL of precooled extraction solution, containing 0.05 mol/L phosphate buffer solution (pH 7.8) and 1% polyvinylpyrrolidone. Then, tissue homogenates were chilled to 4 °C until used for further processes. H_2_O_2_ concentrations were measured using the Hydrogen Peroxide Assay Kit (Beyotime Biotechnology Inc, Shanghai, China) following manufacturer’s instructions. Absorbance was read at 560 nm using a microplate reader (PerkinElmer Inc., Waltham, USA). H_2_O_2_ concentrations were calculated according to a standard concentration curve and expressed as nmol/ml. MDA contents were measured using a Plant Malondialdehyde assay kit (Nanjing Jiancheng Bioengineering Inst., Nanjing, China), which was based on the reaction of thiobarbituric acid (TBA) with MDA. The absorbance of red TBA-MDA complex was read at 532 nm using a microplate reader and the MDA content (nmol/g) was calculated according to manufacturer’s instructions.

### Analysis of *Zm*CAT mRNA expression by quantitative real-time PCR (qRT-PCR)

Total RNA was extracted from leaf samples and assessed as described above. Raw RNA (0.5 µg) was digested using RNase-free DNase I and then reverse-transcribed to cDNA using an AMV First Strand cDNA Synthesis Kit (Sangon Biotech Inc., Shanghai, China). The resulting cDNA samples were analyzed and quantitative PCR was performed in a Bio-Rad iQ5™ Multicolor Real-time PCR Detection System (Bio-Rad Laboratories Inc., Hercules, CA, USA), according to manufacturer’s instructions. The qPCR analysis was performed in a total liquid volume of 25.0 µL, containing 1 × SYBR Green Master Mix, 4.0 µL of the diluted cDNA templates (*n* = 5), 0.4 mM of each primer and 7.5 µL of DEPC-treated RNA-free water. A 140 bps product was amplified with *Zm*-CAT-qRT F and *Zm*CAT-qRT-R (Table S1) and sequenced to verify PCR specificity. The housekeeping gene eukaryotic initiation factor 4A (eIF4A) was used as an internal control to check the RT-qPCR reaction (Ransbotyn & Reusch, 2006). Primers of the internal control gene used for RT-qPCR were listed in Table S1 and produced a fragment of 125 bps. Conditions for the reactions were 95 °C for 3 min, followed by 40 cycles of 95 °C for 15 s, and then 60 °C for 40 s. A melting curve was generated for each sample at the end of the RT-qPCR reaction to confirm the purity of the amplified products. Data from RT-qPCR were analyzed using the 2^−ΔΔCt^ method of the ABI 7500 software 2.0.6 (Applied Biosystems, Inc., Foster City, USA).

### Recombinant overexpression and purification of *Zm*CAT

The pEASY-E1 vector was used for high level, inducible expression of *Zm*CAT recombinant proteins, as has been reported by Liu et al. (2016). In brief, the cDNA fragment encoding the mature peptide of *Zm*CAT was obtained after PCR amplification with the primers, *Zm*CAT-recombinant-F and *Zm*CAT-recombinant-R (Table S1). The plasmid pEASY-E1/*Zm*CAT was isolated by E.Z.N.A.^®^  Plasmid Mini Kit (Omega Bio-Tek Inc., Norcross, GA, USA) and then transformed into *E. coli* strain BL21 (DE3) to produce recombinant protein. The resulting strain was grown at 37 °C in liquid SOB medium coupled with ampicillin (100 mg  mL^−1^) until the OD_600_ reached 0.4–0.6. The bacteria were induced for protein expression by IPTG addition to 1 mM. After incubation for 4 h at 30 °C, the bacteria were harvested by centrifugation and the recombinant protein purified by MagExtractorTM His Tag Kit (Toyobo Co Ltd., Osaka, Japan) under denaturing conditions (8 mol L^−1^ urea). The purified protein was next refolded in a gradient urea-TBS glycerol buffer and the resultant protein mixed 1/1 (wt/wt) with 2× Coomassie blue loading dye. Each sample was loaded on a 12% SDS-PAGE gel and Precision Plus Protein™ standards (Bio-Rad Laboratories Inc., Hercules, CA, USA) added to provide molecular mass markers.

### Analysis of enzymatic activity of recombinant *Zm*CAT

Enzymatic activities of recombinant *Zm*CAT (r*Zm*CAT) were examined using a Catalase Assay Kit (Beyotime Biotechnology Inc., Jiangsu, China) as previously developed methods (Liu et al., 2016). The purified r*Zm*CAT protein was quantified using an Enhanced BCA Protein Assay Kit (Beyotime Biotechnology Inc, Shanghai, China). The enzymatic activities of r*Zm*CAT protein was characterized by treating protein samples at 5 °C intervals between 0 and 40 °C for 24 h.

### Statistical analysis

The data of mRNA expression and enzymatic activities were normalized to 20 °C. All data were presented as the mean ±SD (*n* = 5). One-way analysis of variance (ANOVA) followed by a Student-Newman-Keuls (S-N-K) multiple comparison method was used to analyze the significance of differences in the SPSS 22.0 software and *p*-values of <0.05 considered statistically significant differences.

## Results

### Cloning and sequence analysis of full-length *Zm*CAT cDNA

**Figure 1:**
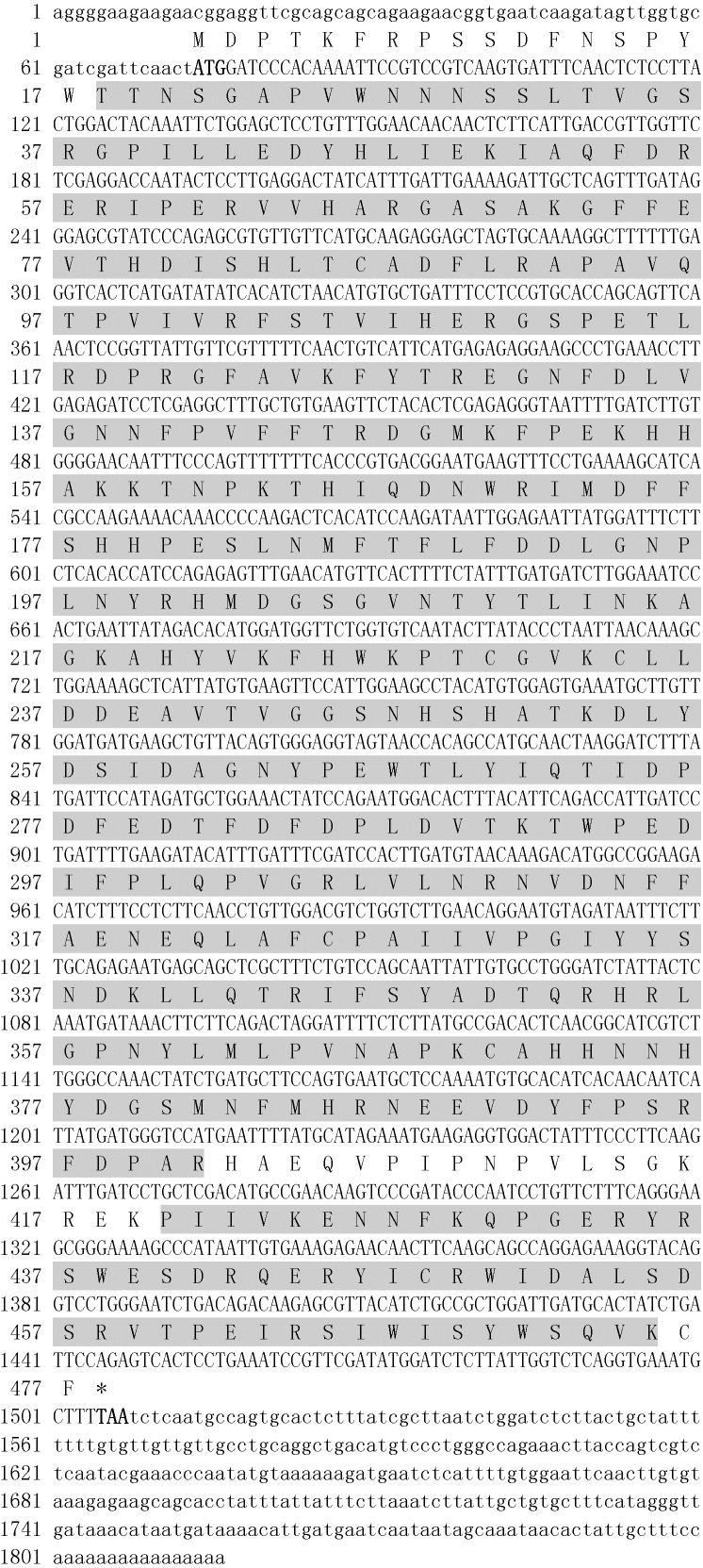
Nucleotide sequence and deduced amino acid sequences of *Zm*CAT catalase. The number of nucleotides and deduced amino acid residues are shown in the left margin. A catalase domain (Thr^18^ to Rrg^401^) and a catalase-rel domain (Pro^420^ to Lys^475^) are shaded. The asterisk indicated the stop codon. The lowercase letters represent nucleotide sequences, and the capital letters indicate amino acid sequences.

The full-length cDNA of *Zm*CAT was obtained by the RACE technique and deposited into GenBank (accession number KJ766310). The complete cDNA of *Zm*CAT consisted 1,816 bp, including a 1,434 bp open reading frame (ORF), 73 bp 5′-untranslated region (UTR), and a 309 bp 3′-UTR (Fig. 1). The 3′-UTR contained a 16 bp poly(A)-tail but no putative polyadenylation signal (AATAAA). The ORF encoded a polypeptide of 477 amino acid residues with a calculated molecular mass of 55.3 kDa, and a theoretical pI point of 6.40. No signal peptide in the deduced amino acid sequence was revealed by SignalP program analysis. Localization prediction software suggested that *Zm*CAT would be localized to the chloroplast. PROSITE analysis showed that the *Zm*CAT amino acid sequence contained highly conserved motifs, which included the proximal active site signature (^54^FDRERIPERVVHARGAS^70^) and proximal heme-ligand signature sequences (^344^RIFSYADTQ^352^). In addition, the His^65^ catalytic residue was found in the active site responsible for proper binding and reduction of peroxide (Fig. 1). The putative *Zm*CAT contained two potential N-glycosylation sites at N-28 (NNSS) and N-247 (NHSH). A CAT core domain at 18–398 (Thr^18^ to Rrg^401^) and CAT immune-responsive domain (Pro^420^ to Lys^475^) were also detected.

### Multiple sequence alignment and phylogenetic analysis of *Zm*CAT

**Figure 2:**
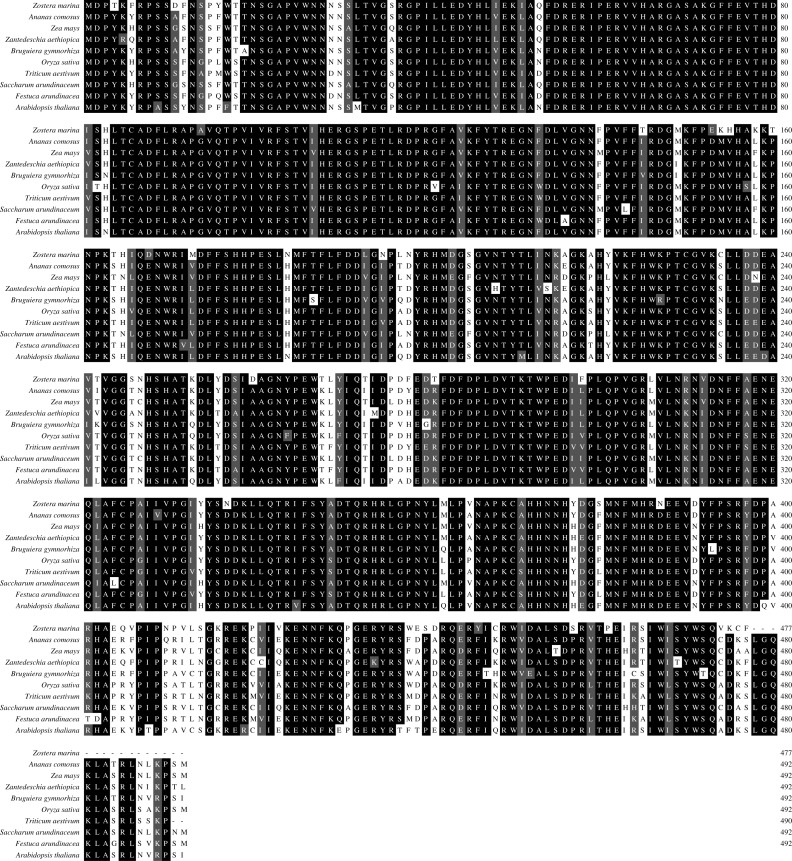
Multiple alignment of deduced amino acid sequences of *Zm*CAT with selected sequences from other plant species. *Ananas comosus* (OAY65449), *Zea mays* (NP_001241808), *Zantedeschia aethiopica* (AAF19965), *Bruguiera gymnorhiza* (ADC95629), *Oryza sativa* (BAA34205) *Triticum aestivum* (AIZ77475), *Saccharum arundinaceum* (AIU99479), *Festuca arundinacea* (CAG23920), *Arabidopsis thaliana* (AAK96854). The black shaded regions represent identical amino acids, while the gray regions represent similar amino acids.

**Figure 3:**
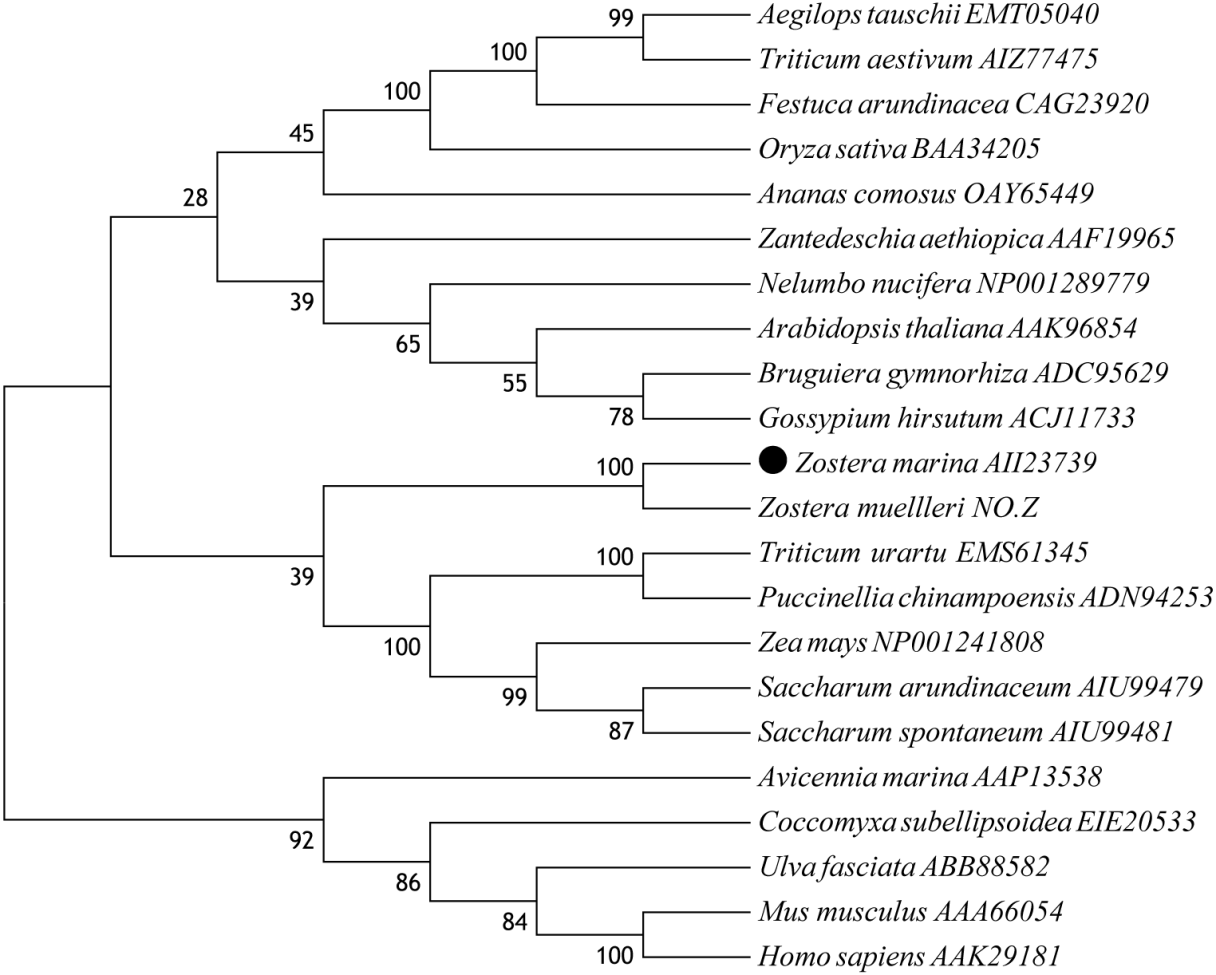
Phylogenetic analysis based on amino acid sequences of *Zm*CAT and other known CATs. Numbers above or below branches at tree nodes indicate percent bootstrap values after 5,000 replicates. The GenBank accession numbers are displayed next to each sequence. Accession NO.Z: The protein sequences of CAT in Zostera mueller was download from http://appliedbioinformatics.com.au/index.php/Seagrass_Zmu_Genome.

A multiple alignment of *Zm*CAT was performed by selecting CAT sequences of nine representative plant species (Fig. 2). The deduced amino acid sequence of *Zm*CAT exhibited high similarity with other previously identified CAT, such as an 88% identity with CAT from *Ananas comosus*, 85% identity with *Zea mays*, 84% identity with CATs from *Zantedeschia aethiopica*, *Bruguiera gymnorhiza*, *Oryza sativa*, *Triticum aestivum*, *Saccharum arundinaceum* and *Festuca arundinacea* and 83% identity with *Arabidopsis thaliana*. The molecular evolutionary relationship of *Zm*CAT was examined by aligning sequences of known CAT members from different taxa and constructing a phylogenetic tree from the conserved regions by the NJ method. The NJ phylogenetic tree was positioned separately into three main branches and *Zm*CAT found to be clustered with CAT from *Zostera muelleri* and located in the angiospermous cluster sub-branch (Fig. 3).

### Effects of temperature stress on the content of H_2_O_2_ and MDA

**Figure 4:**
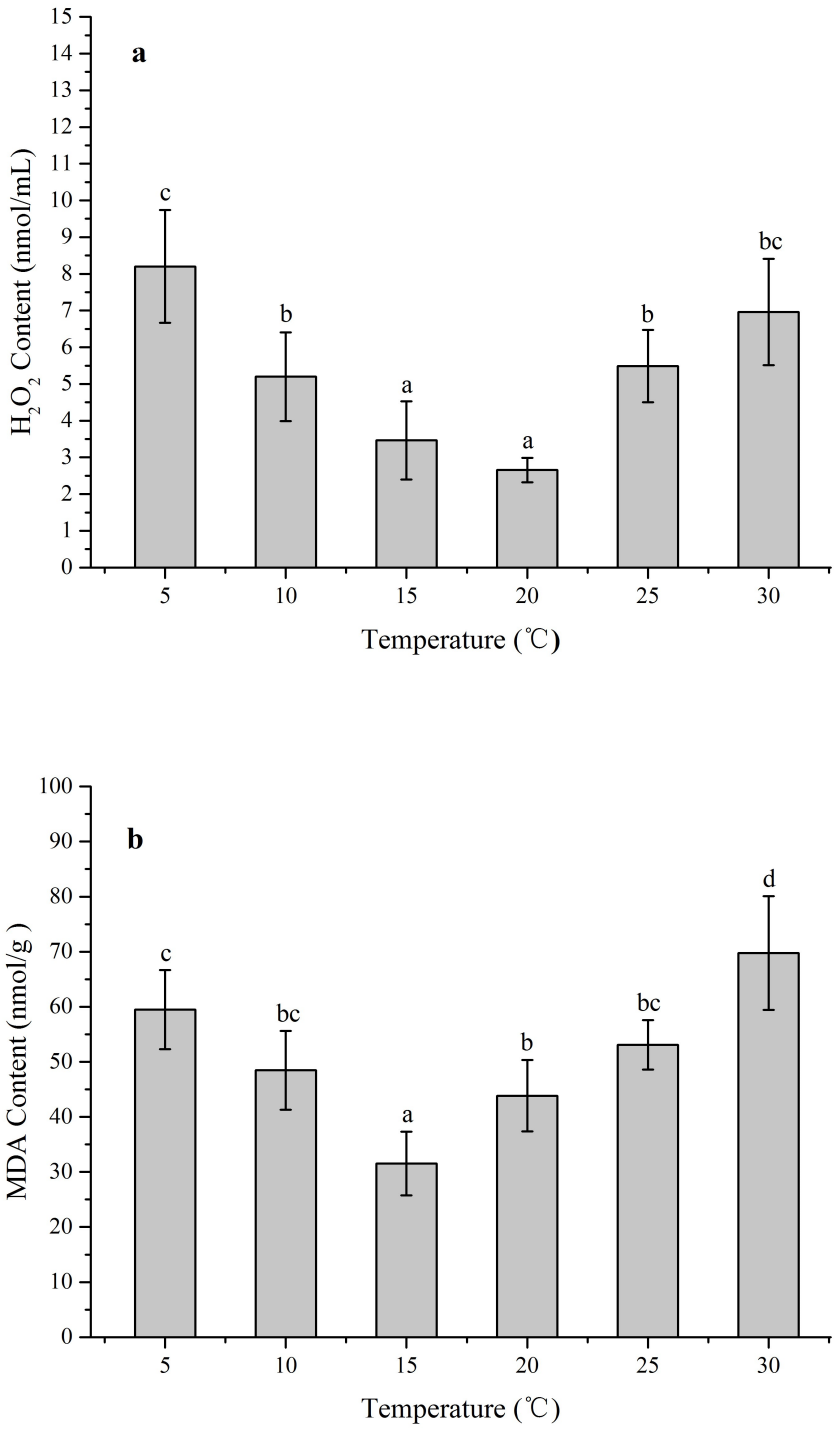
The effects of temperature stress on H_2_O_2_ and MDA contents in *Z. marina* during a 96 h experiment. (A) H_2_O_2_ content. (B) MDA content. Values shown as means ± SD (*n* = 5), bars marked with dissimilar letters represent significant differences (*p* < 0.05) from each other according to S-N-K multiple comparisons tests.

The ROS levels and membrane injury in *Z. marina* under different temperature treatments (5, 10, 15, 20, 25, and 30 °C) after 96 h was investigated in an experiment with different H_2_O_2_ and MDA contents When the temperature decreased from 15 to 5 °C and increased from 20 to 30 °C, the H_2_O_2_ content increased significantly (Fig. 4A). The greatest H_2_O_2_ content increases occurred in the 5 treatment (8.20 ± 1.54 nmol/mL) with 3.1-fold higher than 20 °C (*p* < 0.05), while the H_2_O_2_ content exhibited no significant change and kept at the temperature ranged from 15 to 20 °C. In response to temperature treatments, there were significantly increased MDA contents at the temperature decreased from 15 to 5 °C and increased from 15 °C to 30 °C (Fig. 4B). The highest MDA content occurred in the 30 °C treatments (69.74 ± 10.32 nmol/g) with 2.2-fold higher than 15 °C (*p* < 0.05).

### Effects of temperature stress on *Zm*CAT mRNA expression

**Figure 5:**
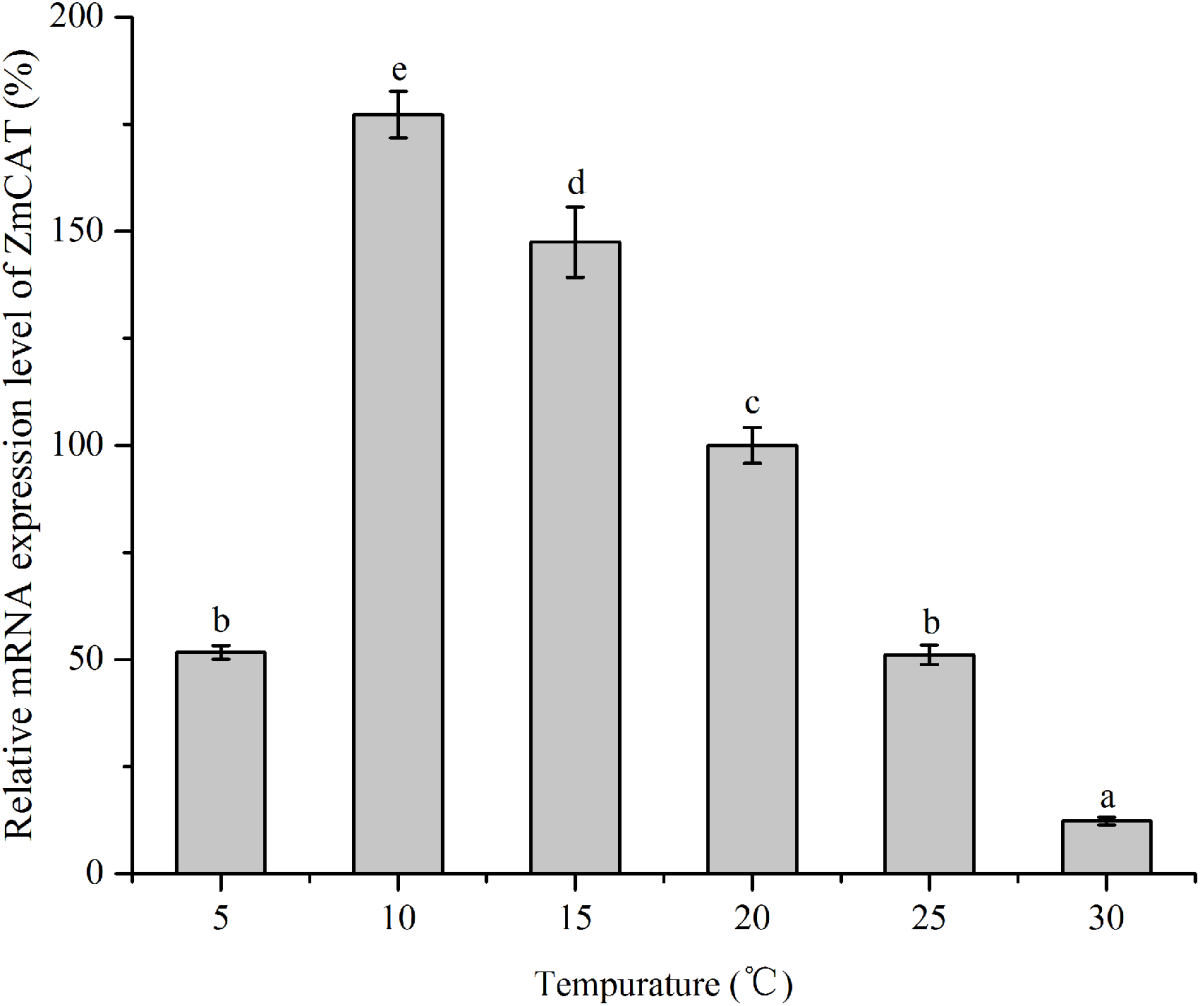
The effects of temperature stress on the relative mRNA expression levels of *Zm*CAT. Amplification of eIF4A gene was used as an internal control. The *Zm*CAT expression levels at 20°C was set to 100%. Values shown as means ± SD (*n* = 5), bars marked with dissimilar letters represent significant differences (*p* < 0.05) from each other according to S-N-K multiple comparisons tests.

RNA levels of CAT gene were quantified using RT-qPCR technique of reverse transcripts of RNA from *Z. marina* that had been subjected to various temperature treatments for 96 h (Fig. 5). The data were then standardized relative to the RNA expression levels of eIF4A, a housekeeping gene consistently expressed in plants. Although relative mRNA expression levels were detected in all treatments, the CAT gene was significantly upregulated from 5 to 15 °C, with the highest expression at 10 °C. In contrast, this gene revealed rather low expression in heat-stressed conditions (25 and 30 °C); and *Zm*CAT expression at 10 °C was 13-fold higher than at 30 °C (*p* < 0.05). Hence, temperature stress suppressed *Zm*CAT expression, but *Zm*CAT was expressed at lower values with elevated heat stress than with cold stress.

### Effects of temperature on recombinant ZmCAT activity

**Figure 6:**
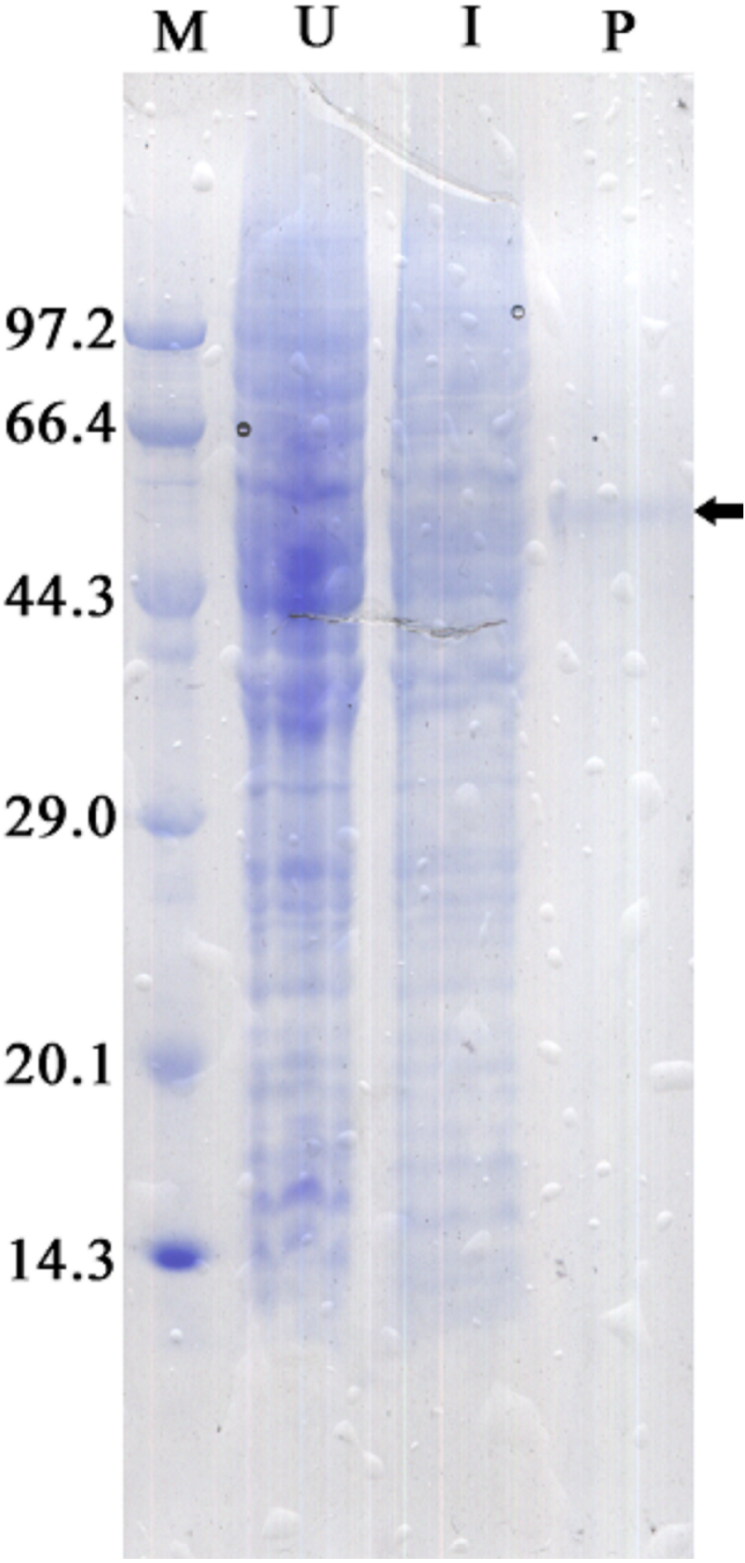
SDS-PAGE analysis of recombinant *Zm.* CAT protein expressed in *E. coli* BL21 (DE3). Samples analyzed included low molecular weight protein markers (M, lane 1), un-induced expression of p*EASY*-E1/*Zm*CAT in total BL21 (DE3) cell lysates (U, lane 2), 1 mM IPTG induced expression of pEASY-E1/*Zm*CAT in BL21 (DE3) (I, lane 3), and the purified recombinant protein of *Zm*CAT (P, lane 4).

**Figure 7:**
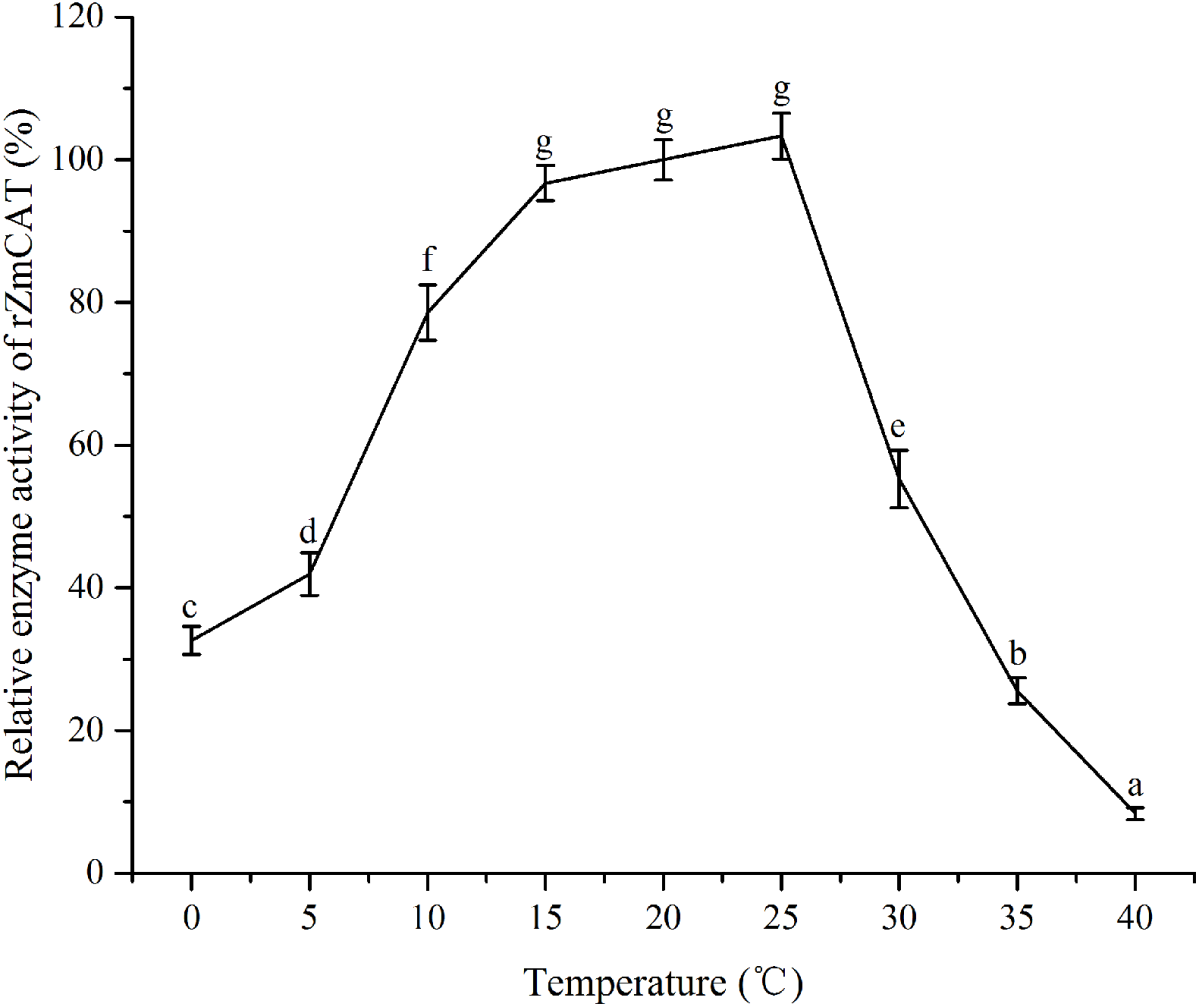
The effects of temperature stress on the enzymatic activity of recombinant *Zm*CAT protein. The enzymatic activity at 20°C was set to 100%. Each values is shown as mean ± S.D. (*n* = 5), and bars with different characters were significantly different (*p* < 0.05).

The recombinant protein activity of *Zm*CAT was determined to examine whether it was functionally active in the antioxidant response under temperature stress. Highly purified *Zm*CAT proteins for biochemical characterization under temperature stress were obtained using *E. coli* BL21 (DE3) as a heterologous expression system to express *Zm*CAT protein and demonstrate *Zm*CAT’s antioxidant activity, based on H_2_O_2_ consumption. Recombinant *Zm*CAT (r*Zm*CAT) protein was analyzed by SDS-PAGE, appearing as a distinct band at a molecular weight of ∼55 kDa, which was close to the calculated molecular mass of *Zm*CAT (55.3 kDa, Fig. 6). The enzymatic activity of recombinant protein was 19,310.5 ± 83.5 U mg^−1^ at 20 °C. The stability of r*Zm*CAT was investigated by measuring the enzymatic activities of its recombinant protein under different temperature treatments (0–40 °C). The enzymatic activity increased rapidly from 0 to 15 °C and remained at a high level from 15 to 25 °C, with >95% of the relative enzymatic activity retained in the temperature range of 15–25 °C. After reaching peak activity at 25 °C the r*Zm*CAT gradually lost enzymatic activity and almost devitalized at 40 °C. It was noteworthy that, although the relative activity was clearly reduced from 25 to 40 °C, >20% of its activity was retained after incubation at 35 °C for 96 h (Fig. 7).

## Discussion

Catalase plays an important role in the antioxidant defense system by degrading the H_2_O_2_ to H_2_O and O_2_ ( Unwin, 1975). In recent years, several CAT genes have been identified in different studies (Du, Zhou & Huang, 2013; Lin, Huang & Lin, 2010), but the present study was the first to deal with CAT cloned and characterized from eelgrass. *Zm*CAT was found to possess most of the main characteristic amino acid residues, motifs, and elements of the CAT protein family. The sequence and multiple alignment results indicated that the active site signature (^54^FDRERIPERVVHARGAS^70^) and heme-ligand signature motifs (^344^RIFSYADTQ^352^) were conserved in *Zm*CAT (Fig. 1). Multiple alignments revealed high sequence identity with CATs from other known plant species, ranging from 83 to 88% (Fig. 2). Phylogenetic relationship collectively suggested that *Zm*CAT was grouped within the angiosperms and might have similar functions to CATs from other plant species (Fig. 3). All present results showed that *Zm*CAT was a representative member of the plant CAT family.

Temperature stress usually causes injury such as excessive accumulation of ROS in plant cells, which seriously damage cell membranes, protein, and nucleic acids (Gill & Tuteja, 2010; Vighi et al., 2016). Low temperature stress can induce destructive oxidative processes leading to an increase of MDA content in tissue, which could reflect the degree of LPO and structural integrity of plant membranes (Li et al., 2013). Therefore, redox states and membrane integrity responses of *Z. marina* exposed to temperature stress were identified by investigating the effects of such stress on H_2_O_2_ and MDA contents. In response to temperature stress, H_2_O_2_ and MDA contents in *Z. marina* significantly increased at cold stress (<10 °C) and heat stress (>25 °C) and the highest H_2_O_2_ and MDA content respectively occurred at 5 and 30 °C (Fig. 4). The observed increased H_2_O_2_ and MDA contents were indications that *Z. marina* was unable to suppress oxidative stress under extreme temperature stress, which might influenced its physiological functions and survival.

The expression profile of CAT was a key part of the plant defense system against oxidative stress (Bi et al., 2017). In the present study, *Zm*CAT appeared to be regulated at the transcriptional level for detoxifying increased H_2_O_2_ during cold stress conditions. The optimal temperature for *Z. marina* growth has been reported to be 15.3 ±1.6 °C (Lee, Sang & Kim, 2007), while the expression concentration increased rapidly from 5 to 10 °C and the highest *Zm*CAT gene expression observed at 10 °C (Fig. 4). Cold stress can lead to excess H_2_O_2_ accumulation and increased H_2_O_2_ content might act as signals for inducing antioxidant gene expressions. Therefore, it was deduced that the *Zm*CAT gene was induced and upregulated to reduce oxidative damage under lower temperature stress, but sustained extreme low temperature stress (5 °C) damages tissue and leads to decreased *Zm*CAT expression and increased H_2_O_2_ content and membrane injury Similar results have been obtained by Baek & Skinner (2003), who found that CAT gene expression was significantly higher in winter wheat after cold-stress exposure. However, *Zm*CAT gene expression was suppressed when the temperature increased from 25 to 30 °C, which showed the inhibiting effects of heat stress conditions on *Zm*CAT gene expression that, in turn, might have resulted in intracellular H_2_O_2_ accumulation.

Information on the recombinant enzyme activity of the *Zm*CAT after temperature treatments was helpful for a better understanding of its physiological function. The relative enzymatic activity retained with >95% by the r*Zm*CAT at 15–25 °C. Field surveys and experiments have confirmed that high water temperatures >25 °C in summer heat waves increasingly threatens seagrass performance and >30 °C can be lethal (Ehlers, Worm & Reusch, 2008; Kaldy, 2014; Nejrup & Pedersen, 2008). However, the highest H_2_O_2_ removal activity by r*Zm*CAT was observed at 25 °C. Moreover, a relative activity of 55% was retained by r*Zm*CAT at 30 °C (Fig. 6). On the basis of its strong antioxidant activity at high temperatures, *Zm*CAT was suggested here to play a role against heat stress, as a potent ROS-detoxifying enzyme in eelgrass. Several CATs have been demonstrated to have high thermal stability, including *Pyropia yezoensis* CAT (*Py* CAT), *Oryza sativa* CAT-A (*Os* CatA), and CAT-C (*Os* CatC), with reported optimal temperatures of ∼30 °C (Li et al., 2012; Vighi et al., 2016). In addition, r*Zm*CAT exhibited higher heat sensitivity than that reported for *Py* CAT. The r*Zm*CAT activity declined rapidly as temperature exceeded 25 °C and <10% activity was retained at 40 °C, similar to that reported for *Os*CatC. A potential reason for this difference might have been because CAT in *Z. marina* and *P. yezoensis* must cope with different living environments, with *P. yezoensis* living in the upper intertidal zone with high and variable temperature, while *Z. marina* is a subtidal population and in a more stable environment. Thus, a more stable enzyme is generated in *P. yezoensis* as an adaptation to harsh living conditions. It should be noted that the increased H_2_O_2_ and MDA content at extreme temperature stress (5 °C and 30 °C) might have resulted from both *Zm*CAT gene expression and enzymatic activity, which were significantly inhibited. Interestingly, *Zm*CAT expression characterization was different from enzyme activity under heat stress. *Zm*CAT mRNA expression was downregulated after 25 and 30 °C temperature treatments for 96 h, while the enzymatic activity first increased and then declined. *Zm*CAT appeared to respond more sensitively at the transcriptional level than at the enzymatic level. These results indicated that *Zm*CAT in eelgrass survived under heat stress through elevated enzymatic activity rather than upregulated gene transcription to protect cellular components against ROS effects produced as a consequence of oxidative stress. This information might be valuable for further analysis of the profile and functional diversity of CAT.

## Conclusions

In this study, a novel CAT gene, named as *Zm*CAT, was identified in *Z. marina* and analysis of its deduced amino acid sequence showed typical CAT functional domains, including the CAT proximal heme-ligand signature and CAT proximal activity site. Multiple sequence alignment as well as phylogenetic analysis provided evidence for the evolutionary conservation of *Zm*CAT, which was a typical member of the plant CAT family. Temperature stress increased oxidative stress and membrane injury in *Z. marina*. To counter this stress, *Zm*CAT presented an antioxidant defense and protection by high enzymatic activity under heat stress and by upregulated gene transcription under cold stress. However, both *Zm*CAT gene expression and enzymatic activity were significantly suppressed at extreme temperatures (5 and 30 °C) which indicated that the ability of *Zm*CAT to scavenge H_2_O_2_ was weakened and led to changes in the cellular redox state and influenced its physiological function in eelgrass. Gene expression and enzymatic activity of *Zm*CAT varied under different temperature stresses, which suggested that *Zm*CAT might have played a central role in mitigating oxidative damage under temperature stress.

##  Supplemental Information

10.7717/peerj.4532/supp-1 Table S1 Specific primers used in the study

## Additional Information and Declarations

### Field Study Permissions

The following information was supplied relating to field study approvals (i.e., approving body and any reference numbers): The research permit was issued to the College of Marine Life Science, Ocean University of China.

### DNA Deposition

The following information was supplied regarding the deposition of DNA sequences. The catalase gene sequences of *Zostera marina* described here are accessible via GenBank accession number KJ766310.

### Data Availability

The following information was supplied regarding data availability: NCBI databases. GenBank: KJ766310.
