# Supplementary material for: Description of a Zostera marina catalase gene involved in responses to temperature stress
Source: PeerJ. 2018 Mar 26;6:e4532. doi: 10.7717/peerj.4532 (PMC5875394; doi:10.7717/peerj.4532)
Supplement: Table S1 [file peerj-06-4532-s001.docx]

Table S1. Specific primers used in the study

| Primer name | (5'to3') nucleotide sequence |
| --- | --- |
| oligo dT-adaptor  *Zm*CAT-Race-F1  *Zm*CAT-Race-F2  M13-47  RV-M  *Zm*CAT-qRT-F  *Zm*CAT-qRT-R  eIF4A- RT-F  eIF4A- RT-R  *Zm*CAT-recombinant-F  *Zm*CAT-recombinant-R  T7 promoter primer  T7 terminator primer | GGATCCGAATTCCCCGGG(T)24  GGCTTGCTTATGACAAAGAGTTGAAGA  TGGAAATACGCCAGCCAGGTCTAC  CGCCAGGGTTTCCCAGTCACGAC  GAGCGGATAACAATTTCACACAGG  CCCAAGACTCACATCCAAGA  TTGACACCAGAACCATCCAT  CCCAAGACTCACATCCAAGA  TGGATGTATCGGCAGAAACG  ATGTCGCTCTTGGTTGATCCTTGTTGT  TTAAGCAGATTCTTTCTTGTAGACCTG  TAATACGACTCACTATAGGG  TGCTAGTTATTGCTCAGCGG |

|  |  |
| --- | --- |
